# Impact of conjugation strategies for targeting of antibodies in gold nanoparticles for ultrasensitive detection of 17β-estradiol

**DOI:** 10.1038/s41598-019-50424-5

**Published:** 2019-09-25

**Authors:** Jairo Pinto Oliveira, Adilson Ribeiro Prado, Wanderson Juvencio Keijok, Paulo Wagnner Pereira Antunes, Enrique Ronald Yapuchura, Marco Cesar Cunegundes Guimarães

**Affiliations:** 1 Federal University of Espirito Santo, Av Marechal Campos1468, Vitoria, ES 29.040-090 Brazil; 2 Federal Institute of Espírito Santo, km 6.5 ES 010, Serra, ES 29173-087 Brazil; 3 Federal University of Espirito Santo, Av. Fernando Ferrari, Vitoria, ES, 29075-910 Brazil

**Keywords:** Biosensors, Nanoparticles

## Abstract

Antibody-coated nanoparticles have recently attracted considerable attention, with the focus falling on diagnostics. Nevertheless, controlled antibody bioconjugation remains a challenge. Here, we present two strategies of bioconjugation with the aim of evaluating the best approach for the coupling of antibodies on the surface of nanomaterials in an oriented way. We employed electrostatic interaction (physical adsorption) and covalent conjugation in the orientation of antibodies on the metallic surface as coupling methods, and their influence on the detection of 17β-estradiol was addressed with localized surface plasmon resonance. The understanding of these mechanisms is fundamental for the development of reproducible inorganic bioconjugates with oriented surface as well sensibility of immunoassays.

## Introduction

Due to their high specificity and great diversity, antibodies (Ab) are widely used to provide specificity and bioactivity to nanoparticles (NPs), mainly for biosensor applications, drug delivery and imaging marking^1–6^. Thanks to advances in biotechnology, it is now possible to produce antibodies on a large scale in various biological systems against practically any target^7^. However, despite the importance of these molecules to nanoparticulate systems, the preparation and optimization of reproducible bioconjugates are limiting factors for their progress in biotech applications.

Numerous physico-chemical methods have been proposed to couple and functionalize several types of NPs with antibodies^8–25^. One such method involves ionic adsorption occuring at a complexing pH near the isoelectric point (pI) of the antibody. Despite having disadvantages such as poor reproducibility, random orientation and low stability at different pH conditions, Puertas *et al*.^26^ reported good results in the orientation of antibodies with this approach. There is evidence that the plane of interaction of the antibodies with the metal surface does not affect the antigen binding portion (Fab), due to the net charge distribution and the asymmetry of the antibody.

Another method of bioconjugation involves the modification of the surface of nanoparticles with reactive groups such as carboxyl and amine groups, which can be covalently coupled to amino acid side chains on the surface of the antibody using standardized bioconjugation methods such as EDC/NHS^27^. Although this is the most widely diffused method, due to high stability and covalent bonding to the metal surface, some authors have reported aggregation and polymerization^28^. In addition, there may be random orientation of the antibody on the surface of the nanoparticle, which could affect the accessibility of antigen binding sites^17,26,29^.

Thus, we sought to investigate the best approach regarding conjugation efficiency and orientation of the antibodies on the metal surface using the two strategies discussed above. Bioconjugation assays were performed with anti-17β-estradiol IgG antibodies. Ligated binding was determined by fluorescence using a secondary antibody anti-Fab Alexa Fluor 750 and by transmission electron microscopy using immunogold anti-Fab. Finally, we quantitatively analyzed the efficiency of the nanoconjugates in the detection of 17β-estradiol by localized surface plasmon resonance (LSPR) and high performance liquid chromatography (HPLC).

## Experimental

### Materials

Tetrachlorouronic acid (G4022), trisodium citrate (PHR1416), 16-mercaptohexadecanoic acid (MUA) (448303), polyethylene glycol (PEG) (729159), 17β-estradiol (E-2257) n-hydroxysuccinamide (NHS) (130672) and 1-ethyl-3- (3-dimethylaminopropyl) carbodiimide (EDC) (E6383) were of analytical purity and purchased from Sigma Aldrich. The primary antibody of the IgG1 anti-17β-estradiol type (ab20626) and the fluorophore-labeled secondary antibody (Alexa Fluor 750) anti-Fab IgG1 (ab175740) were purchased from Abcam. For the cleaning of glassworks, we employed a solution of HNO3 and HCl (3: 1) in fresh water and then used it within 1 hour.

### Instruments

Optical properties were evaluated by UV-vis spectrophotometry (FEMTO 800 XI). The size and morphology of the gold nanoparticles (AuNPs) were examined by transmission electron microscopy (TEM) using a JEOL microscope, model JEM1400 operated at 120KV with lanthanum hexaboride (LAB6) filament. The crystalline nature of AuNPs was confirmed by X-ray diffractometry (XRD) using the D8-ADVANCE Diffractometer (BRUKER-AXS). The total concentration of nanoparticles was determined using plasma inductively coupled to a Perkin Elmer mass spectrometer (ICP-MS), model Optima 7000, USA. Ligand functionalization (MUA) was confirmed by the absorption in the infrared region (FT-MIR FTLA 2000 Bomem) and Raman spectroscopy (ALPHA 300 R Raman Spectrometer). The Varioskan Flash Fluorescence Detector (Thermo Scientific) was used to quantify primary antibodies (280 nm excitation and emission scanning from 300 nm to 500 nm) and secondary antibodies (excitation at 750 nm and emission scanning from 770 to 840 nm). The chromatographic system used to detect 17β-estradiol was the Shimadzu CBM-20A model, consisting of a DGU-20AS degasser, an LC-20AT pump, a SIL-20AHT automatic injector and a CTO-20A furnace. Ultrapure water for all tests and preparation of solutions was obtained from the EASYpure II® Thermo Scientific ultrapurification system.

### Softwares

Origin Pro 8.5 free version and GraphPad Prism version 6.01 were used for the elaboration of UV-Vis, fluorescence and HPLC graphs as well as for statistical analysis. Chemdraw Prime software (courtesy of Perkin Elmer) was used to create schemas throughout the text.

### Synthesis and characterization of AuNPs

Synthesis was performed according to the optimization for size control already described by our group^30^. Briefly, 15 mL of 2.5 × 10^−4^ M HAuCl_4_ were allowed to react with 1 mL of 1% sodium citrate for 15 minutes at 100 °C. Afterwards, the colloid was cooled in an ice bath and subjected to centrifugation (16.873 g, 20 min) (MiniSpin 5418, Eppendorf). Three washing steps were performed to remove the unreacted substances. The material was characterized by UV-Vis, TEM and XRD.

### Conjugation of AuNPs with antibodies

For the conjugation of antibodies on the metal surface of AuNPs two strategies were evaluated: (i) direct conjugation of the antibodies by electrostatic attraction; (ii) modification of the metal surface of the NPs for the covalent coupling of antibodies.

#### Direct conjugation of antibodies by electrostatic attraction

The NPs were washed and resuspended in ultrapure water with pH adjusted to the complexing pH (8.5), which is the pI of an IgG-like protein. After this step, 2.8 μg of the anti-17β-estradiol antibody was added to 100 μL of the gold colloid and maintained under orbital shaking at 100 rpm at 25 °C for 30 minutes. Finally, the material was centrifuged (16.873, 20 min) and the pellet was resuspended in 100 μl PEG (1%) dissolved in ultrapure water.

#### Modification of the metallic surface of the NPs for the covalent coupling of antibodies

The NPs were functionalized with the thiolated mercaptohexadecanoic linker (MUA), with 11 carbon atoms between the COOH and SH groups. For AuNPs functionalization, 100 μL of 10 mM MUA was added to 1 mL of AuNPs for 100 h at 800 rpm at 25 °C. The modified nanoparticles were washed 3× by centrifugation (16.873 g, 20 min) and then resuspended in ultrapure water. Bioconjugation was performed using the EDC/NHS method, whose experimental conditions were previously optimized by our research group. Briefly, 100 μL of the ligand-modified surface nanoparticles (MUA) was added to 10 μL EDC (50 μM) for 30 minutes. Next, 10 μL of NHS (75 μM) was added to the mixture, which was left under orbital agitation for 30 minutes at 150 rpm. Subsequently, 2.8 μg of the anti-17β estradiol antibody was inserted into the system and allowed to react for 30 minutes. The antibody was immobilized through its amino groups, forming a pre-activated carboxylic acid amide bond (EDC/NHS). Finally, the material was centrifuged (16.873 g, 20 min) and the pellet was resuspended in 100 μl of 1% PEG for functionalization of the non-antibody coated areas.

### Determination of total bound antibodies

The amount of antibodies bound to the nanoparticles was calculated by subtracting the initial concentration from the free protein detected in the supernatant by fluorescence (excitation at 280 nm and emission at 350 nm).

### Determination of the orientation of bound antibodies

To determine the orientation of the antibodies, we used a fluorophore-labeled (Alexa Fluor 750) anti-IgG Fab secondary antibody. For this step 4 μg of this antibody was added to 100 μL of each of the two types of nanobioconjugates evaluated, 1.84 μg antibodies/AuNPs (mL) for direct conjugation by electrostatic attraction and 1.22 μg antibodies/AuNPs (mL) for covalent conjugation. The amount of non-oriented vs -directed antibodies on the surface of the NPs was calculated by subtracting the initial concentration from the free secondary antibodies in the supernatant by fluorescence (excitation at 750 nm and emission at 770 nm).

### Detection of 17β-estradiol by LSPR

To analyze the sensitivity of the developed gold immunoconjugates (physical adsorption and covalente attachment) based LSPR, the 17β-estradiol was diluted serially (100, 200, 500, 1000, 2000, 5000, 10000 and 20000 ng.mL^-1^) and incubate with 100 uL of Ab-AuNPS separately for 5 min to achieve the proper binding of imunoconjugate and the 17β-estradiol. The LSPR peak intensity was then measured using a UV/Vis spectrophotometer (400 to 700 nm). The detection sensitivity of the developed assay was determined based on the LSPR peak intensities. All experiments were repeated at least three times.

### Detection of 17β-estradiol by HPLC

For chromatographic analysis, 17β-estradiol was used as a reference standard. Peaks were separated by an increasing gradient of acetonitrile at a flow rate of 0.50 mL/min. Hormone levels were determined based on the analytical curve relating area and concentration. Separation of the analyte was performed on a Kinetex TM C18 analytical column (100 × 2.1 mm, 2.6 μ, 100 Å) at 45 °C. The mobile phases consisted of MilliQ water (A) and acetonitrile (B) previously filtered on PTFE membranes (0.45 μm, 47 mm, Millipore) and degassed in an ultrasonic bath (Limpsonic®). The injection volume was 10 μL and detection was performed by PDA diode array detectors (SPD-M20A) at a wavelength of 280 nm.

## Results and Discussion

### Synthesis and characterization of AuNPs

**Figure 1:**
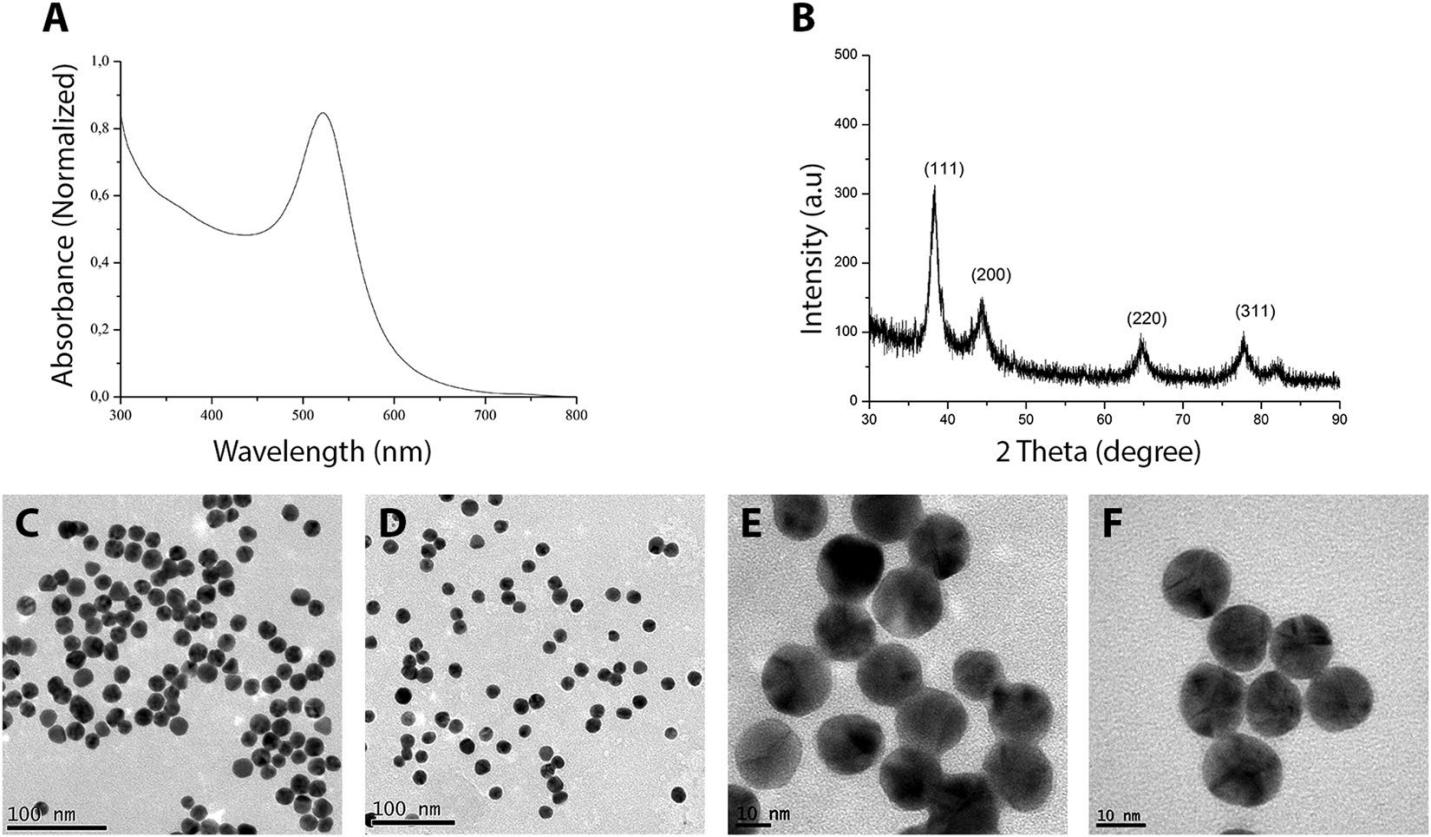
UV-Visible absorption spectroscopy for synthesized gold colloid showing the characteristic plasmony peak at 522 nm (**A**) X-ray diffraction pattern depicting the characteristic peaks of nanocrystals. (**B**) Images obtained by transmission electron microscopy at different magnifications showing the size and distribution of the synthesized nanoparticles. Scale bar 100 nm (**C**,**D**) and 10 nm (**E**,**F**).

AuNPs synthesized with sodium citrate were monodisperse, with a mean diameter of 18 nm (CV < 10%) according to the count of 500 particles obtained by transmission electron microscopy. The characteristic plasmony peak can be observed at 522 nm in the optical absorption spectrum (Fig. 1A), with the narrow bandwidth indicating a small size variation in the synthesized material. The formation of the nanocrystals was also confirmed by X-ray analysis (Fig. 1B), with a more pronounced peak (111) at 38.31° indicating their predominant orientation. Both size distribution and spherical shape can be visualized in Fig. 1(C–F). The concentration determined by ICP-MS was 27.4 mg/L Au.

#### Nanoparticles functionalized with MUA for covalent coupling

**Figure 2:**
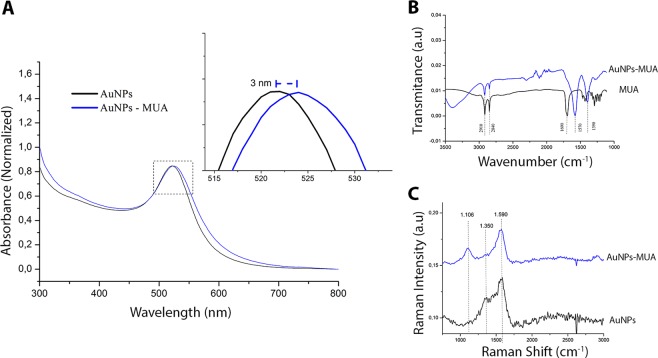
Normalized UV-Vis absorption spectroscopy of AuNPs in black and AuNPs functionalized with mercaptoundecanoic acid (MUA) in blue. (**A**) The inset shows the band displacement caused by the MUA connection with the metal surface; Infrared spectrum of the binder used (MUA) and the functionalized AuNPs showing the overlapping characteristic peaks (**B**). Raman spectra of AuNPs (black) and AuNPs-MUA (blue) (**C**).

Metal surface modification of the NPs was performed for covalent coupling of antibodies using the mercaptoundecanoic acid binder (MUA). The functionalization of the AuNPs with this ligand was evaluated by optical absorption (UV-Vis), absorption in the infrared region and Raman scattering, as can be seen in details in Fig. 2.

The UV-Vis spectra (Fig. 2) shows the plasmonic peak of the monodisperse AuNPs around 521 nm and the AuNPs-MUA peak with bathochromic shift (524 nm). This displacement indicates the functionalization of the metal nanoparticles by the ligand (MUA). Infrared spectra (Fig. 2B) of MUA and AuNPs-MUA showed peaks at 2840 and 2910 cm^−1^ which correspond to asymmetric and symmetrical C-H elongation vibrations respectively. The displacements of the 1390 and 1570 cm^−1^ peaks relative to the control are characteristic of carboxylic acid (COO^−1^) vibrations and suggest the coordination of the ligand with the metal. The Raman spectrum (Fig. 2C) shows AuNPs-MUA with peak 1106 cm^−1^ corresponding to S-C bonds. The 1350 cm^−1^ control peak (AuNPs) corresponds to an asymmetric elongation vibration and its absence in the AuNPs-MUA suggests that sulfide ions replace part of the citrate ions on the metal surface. The peak around 1571 cm^−1^ refers to symmetrical stretching vibrations.

### Conjugation of AuNPs with antibodies

In order to build a sensitive detection system using antibodies conjugated with AuNPs, a minimum concentration of antibodies adsorbed to the metal surface must be determined. For this purpose, the total amount of antibodies bound to the surface was quantified by the intrinsic fluorescence of the protein, which can be attributed to the combination of the fluorescence emitted by the aromatic amino acids in the antibody protein chain. Excitation was set at 280 nm and emission recorded from 300 to 500 nm an a standard curve was performed at 350 nm, (Supporting Information, Fig. S1).

To confirm that the Fab region of the antibodies was not involved in the immobilization, the orientation of the antibodies bound to the metal surface was evaluated in both conjugation methods tested here. To this end, a secondary antibody capable of recognizing the Alexa Fluor 750 fluorophore-labeled IgG Fab portion was used. All of the Ab-AuNPs preparations were incubated with an excess of the secondary antibody. After washing, the amount of bound secondary antibody was quantified by the fluorescence of the Alexa Fluor 750 probe, with excitation at 745 nm and emission at 770 nm. Thus, the presence of the secondary antibody in the sample can be used to calculate the amount of targeted primary antibody (free Fab portion) during immobilization. Figure S2 (Supporting Information) shows the standard curve for detection of the secondary antibody in the sample.

Another important point to be considered in determining the orientation of the conjugated primary antibodies is that there are three possibilities of interaction with the secondary antibody (Supporting Information, Fig. S3). The different routes can determine the amount of secondary antibodies present on the surface of the sample.

**Figure 3:**
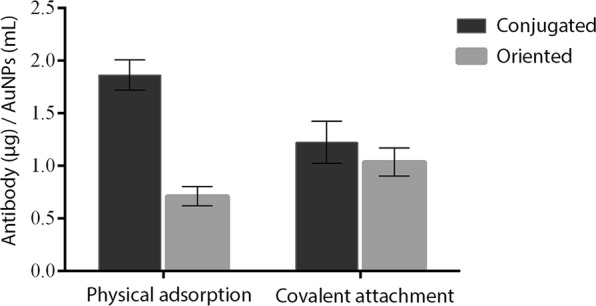
Comparison between the amounts of bound anti-17β-estradiol antibodies and antibodies targeted on the AuNPs metal surface by the conjugation strategies evaluated (Physical adsorption and covalent attachment). The rate of bound antibodies was determined by the intrinsic fluorescence of the amino acids present in the IgG-like antibody structure (Excitation 280/emission 350 nm) and the rate of targeted antibodies was determined by the signal emitted by the anti-Fab Alexa Fluor 750 secondary antibodies.

The physical adsorption conjugation method had a higher rate of antibodies bound in AuNPs, although the orientation rate was only 38.2% (Fig. 3). This result confirms that the Fab regions of many molecules are involved during immobilization, thereby reducing their antigen recognition efficiency. Also, the density of antibodies on the metal surface may influence the availability of the Fab portion: the higher it gets, the less accessibility to antigens should be observed due to steric hindrance between antibodies in close proximity.

As to covalent conjugation, a lower rate of conjugated antibodies was observed (Fig. 3). However, the ratio of targeted antibodies was 84.7%, being significantly higher than what was observed for adsorbed antibodies. This result suggests that conjugation of antibodies by covalent binding promotes better orientation and probably greater sensitivity for antigen-antibody recognition applications.

Transmission electron microscopy was used to qualitatively evaluate the biological activity of the immunoconjugates. This methodology was recently employed by Chou *et al*.^31^ and Fernandez^32^ in order to produce superstructures of nanoparticles for controlled biological delivery and to determine the orientation of antibodies. To this end, anti-Fab secondary antibodies labeled with a 5 nm gold NP (immunogold) were used in order to recognize the available epitopes of the primary antibodies on the metal surface. Figure S4 (Supporting Information) shows an illustration depicting this interaction.

**Figure 4:**
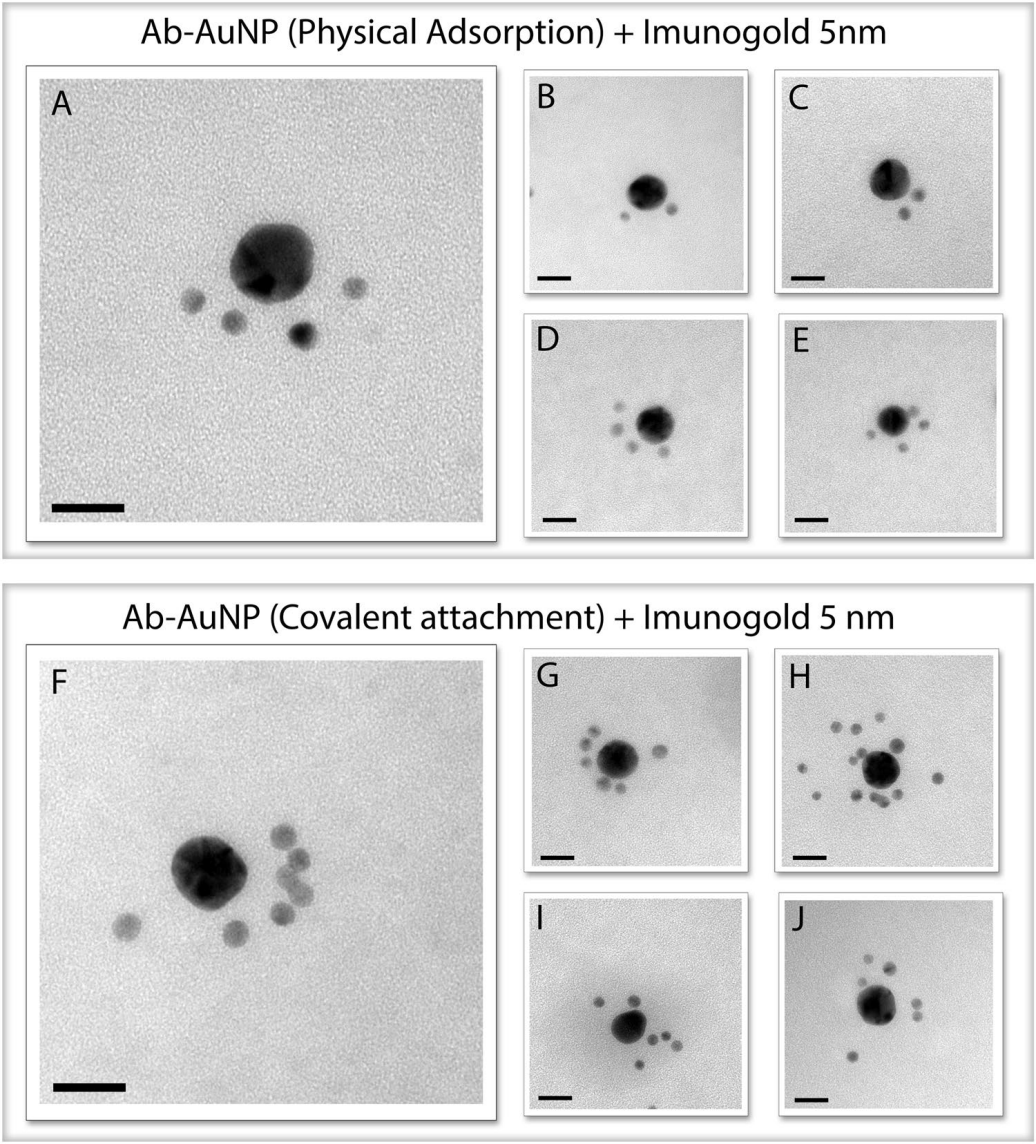
Images obtained by transmission electron microscopy presenting several possible combinations according to the orientation of the primary antibody anchored on the metal surface. Superstructures are formed by recognition of Fab sites by immunogold (5 nm). Immunoconjugates prepared by physical adsorption (**A**–**E**) and immunoconjugates prepared by covalent conjugation through the mercaptoundecanoic acid linker (**F**–**J**). Scale bar 20 nm.

Based on this principle, the bioconjugates produced by the two strategies presented here were evaluated. Structures produced by physical adsorption showed an average of 3 antibodies available per NP (Fig. 4A–E). However, one must keep in mind that, as this is only a qualitative method, other markers may be anchored in the three-dimensional bioconjugate. In covalent conjugation (Fig. 4F–J), a greater number of antibodies available for recognition – approximately 6.5 per NP – was found, confirming the results obtained by fluorescence labeling discussed above. The characterization of these systems by MET revealed the successful formation of superstructures, confirming the recognition between the anti-IgG Fab (Immunogold) and the anti-17β estradiol, as well as proving that the bioconjugates retain their biological activity.

### Detection of 17β-estradiol by LSPR

**Figure 5:**
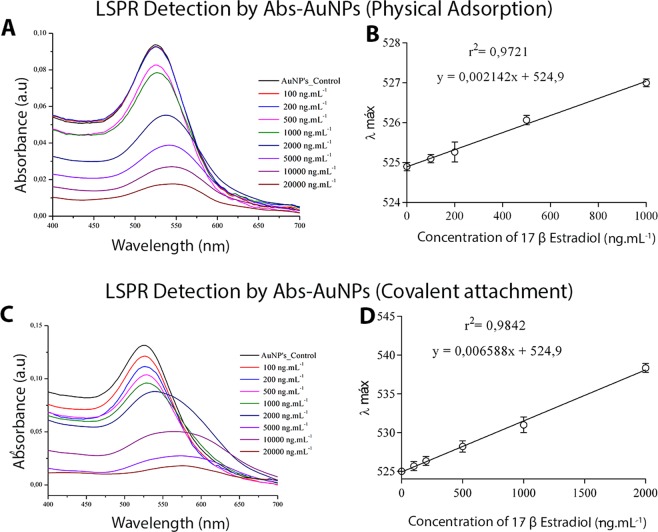
Absorption spectra of the AuNPs conjugated solutions by physical adsorption with anti-17β-estradiol antibodies under different concentrations of 17β-estradiol (**A**); Typical calibration curve for detection of 17β-estradiol with immunoconjugates prepared by physical adsorption. (**B**) Absorption spectra of the AuNPs conjugated by covalent binding with anti-17β-estradiol antibodies under different concentrations of 17β-estradiol (**C**); Typical calibration curve for detection of 17β-estradiol with immunoconjugates prepared by covalent conjugation (**D**).

In order to verify the applicability of the immunoconjugates, as well as their differences in diagnostic assays, the conjugates produced by the two strategies were evaluated for 17β-estradiol detection using surface plasmon resonance. To that end, 100 μL of the colloid containing each of the bioconjugates was incubated with different concentrations of 17β-estradiol for 5 min, having its optical properties evaluated by spectrophotometry (400 to 700 nm). Increasing concentrations of 17β-estradiol led to a red shift of the absorption peak for the conjugates obtained through both strategies, as a result of there being increased amounts of organic molecules anchored to the NPs (Fig. 5). It is interesting to note that, while in the system prepared by physical adsorption changes were first detected at 500 ng.mL^−1^ of 17β-estradiol, for those obtained through covalent conjugation – in which a larger number of targeted antibodies can be found – the detection occurs at 200 ng.mL^−1^. This means that the sensitivity is improved by about 2.5-fold in immunoconjugates produced by covalent attachment. Such increased sensitivity may be attributed to the higher concentration of targeted antibodies, as well as to the creation of more contact points on the surface of the AuNPs. The linear relationship between λ_max_ displacement and 17β-estradiol concentration indicates that the latter can be quantitatively detected.

**Figure 6:**
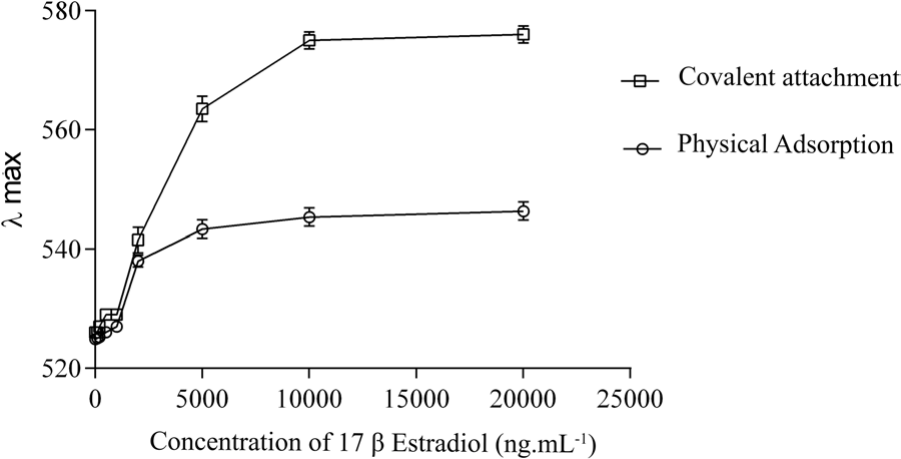
Comparison of the maximum lambda displacement (nm) evolution as a function of increasing concentrations of 17β-estradiol (ng.mL^−1^) for both conjugation strategies evaluated (physical adsorption and covalent attachment).

The evolution of the maximum λ displacement for the two evaluated systems is shown in Fig. 6. For the conjugates produced by physical adsorption, saturation was achieved at about 5000 ng.mL^−1^, half the concentration required to saturate the system synthesized by covalent conjugation. This difference can be attributed to the greater ability of the immunoconjugates prepared by covalent conjugation to interact with the antigen (17β-estradiol), as a result of greater availability of free epitopes for binding. Also, larger red shifts were observed for the covalently linked bioconjugates, which may be due to larger coordination spheres being formed when this strategy was employed.

The detection of the same concentrations of 17β-estradiol was also evaluated by high performance liquid chromatography (HPLC) for comparison with the immunoassays evaluated in this study (Fig. S5, Supporting Information). Although the detection by HPLC showed good linearity (r^2^ = 0.99), lower levels of detection for both immunoconjugates were achieved through LSPR.

The results reported here showed that the orientation of the antibodies on the metal surface can be improved using the covalent binding strategy. Both the ability to recognize the antigen and the biological activity were maintained after the conjugation assays. The low yields observed in the electrostatic adsorption method as to orientation rates suggest that the Fab moieties may be involved – randomly or not – in the conjugation with the metal.

The combination of the localized surface plasmon resonance technique with surface-oriented methods carries a great potential for the development and preparation of affinity biosensors, as shown here for the detection of 17β-estradiol, enabling the real-time analysis of bio-specific interactions without the use of markers. In addition, due to its simplicity, it becomes a potential candidate for the development of miniaturized and portable systems capable of quick and accurate detection of biomolecules.

## Conclusions

The activity of immobilized antibodies in AuNPs is a balance of two factors: density and orientation. The results presented here demonstrate that maximizing the amount of antibodies is not necessarily advantageous as often assumed in the literature. We have also shown that the orientation of the antibodies can be improved using the covalent binding strategy. Finally, we present the applicability of immuconjugates for the detection of 17β-estradiol using LSPR. The proposed methodology can be further improved for use in more sensitive, selective and low-cost nanodevices.

## Supplementary information

Supplementary information
