# Supplementary material for: Impact of conjugation strategies for targeting of antibodies in gold nanoparticles for ultrasensitive detection of 17β-estradiol
Source: Sci Rep. 2019 Sep 25;9:13859. doi: 10.1038/s41598-019-50424-5 (PMC6761283; doi:10.1038/s41598-019-50424-5)
Supplement: Supplementary file 1 — Supplementary information [file 41598_2019_50424_MOESM1_ESM.pdf]

Supporting Information

Impact of conjugation strategies for targeting of antibodies in gold nanoparticles for ultrasensitive detection of 17β-estradiol

Jairo P. Oliveira[1], *Adilson R. Prado*[2], *Wanderson J. Keijok*[1], *Paulo W. P. Antunes*[3], *Enrique R. Yapuchura*[3] *and Marco C. C. Guimarães*[1*]

[1] Federal University of Espirito Santo, Av Marechal Campos1468, Vitória, ES 29.040-090, Brazil

[2] Federal Institute of Espírito Santo, km 6.5 ES 010, Serra, ES 29173-087, Brazil

[3] Federal University of Espirito Santo, Av. Fernando Ferrari, Vitoria ES, 29075-910, Brazil.

* Correspondent Author: *jairo.oliveira@ufes.br / +55 27 3335-7365*

Supplementary Figures

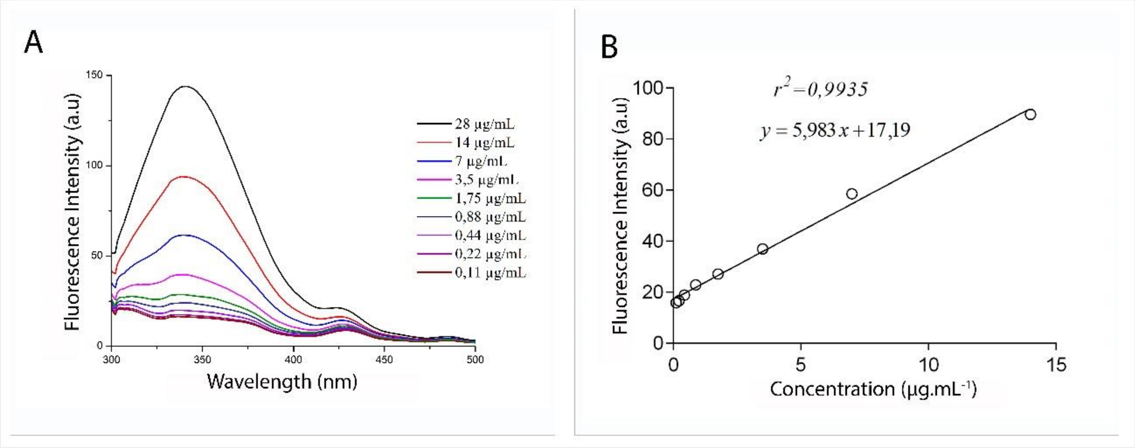

Figure S1 – Fluorescence emission spectra in the detection of the IgG (Anti-17β estradiol) primary antibody molecule with photon excitation performed at 280 nm and emission scanning at 290 to 500 nm (A); Standard curve performed using λ 350 nm (emission) concentrations from 0.11 to 14 µg.mL^{-1} (B).

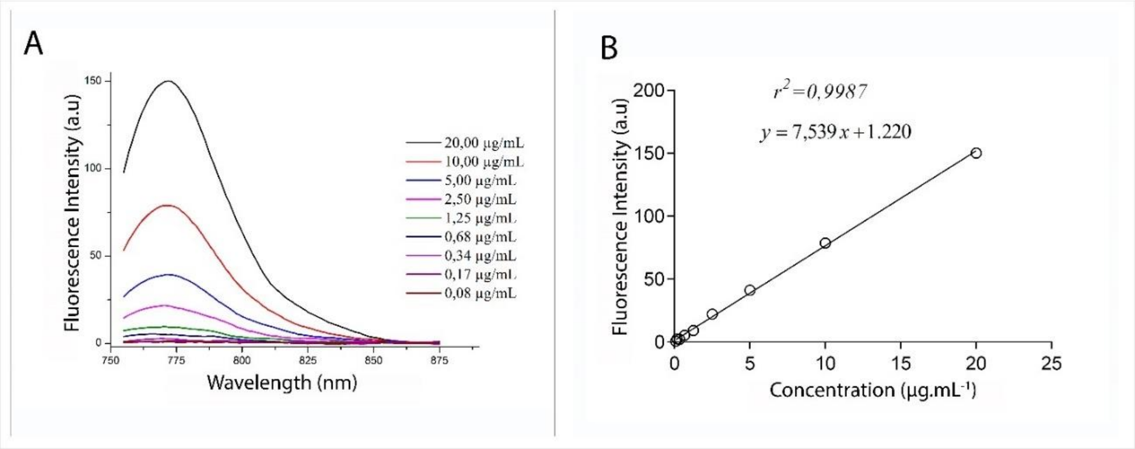

Figure S2 – Fluorescence emission spectra in the detection of the fluorophore-labeled secondary antibody molecule (Alexa Fluor 750) anti-Fab IgG present in the supernatant with excitation at 745 nm and emission scanned from 755 to 875 nm (A); Standard curve obtained at λ 770 nm (emission) with concentrations from 0.08 to 20 µg.mL-1 (B).

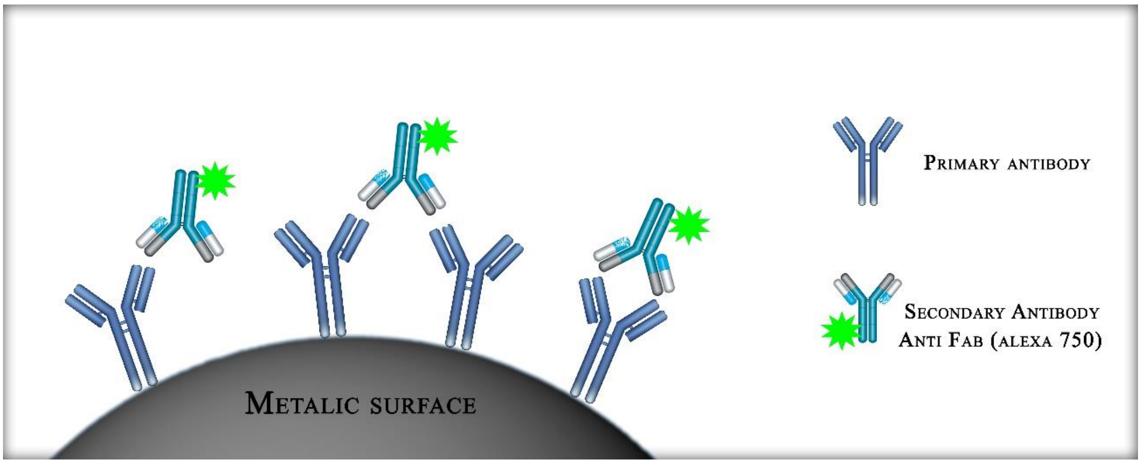

Figure S3. Possibilities for recognition of the Fab portion by the secondary antibody. Antibodies may bind to a site only or to both IgG Fab sites simultaneously, and the primary ab:secondary ab ratio may be found in the 1:1 and 1:2 configurations.

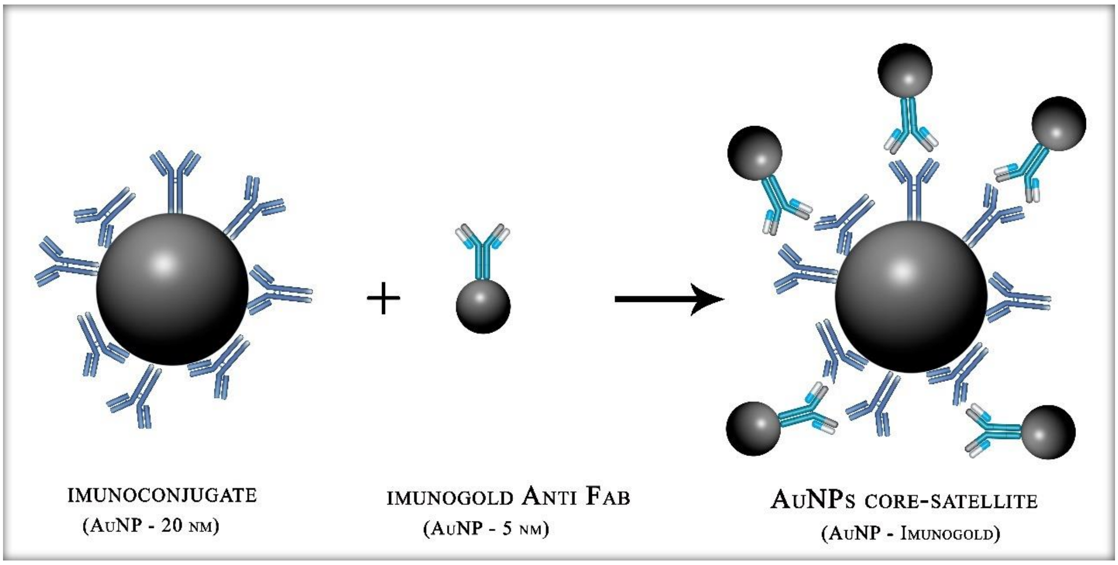

Figure S4. Illustration describing the formation of superstructures with gold nanoparticles involving recognition of the Fab portion of the primary antibody anchored on the metal surface by means of a secondary anti-Fab IgG antibody labeled with 5 nm gold nanoparticles.

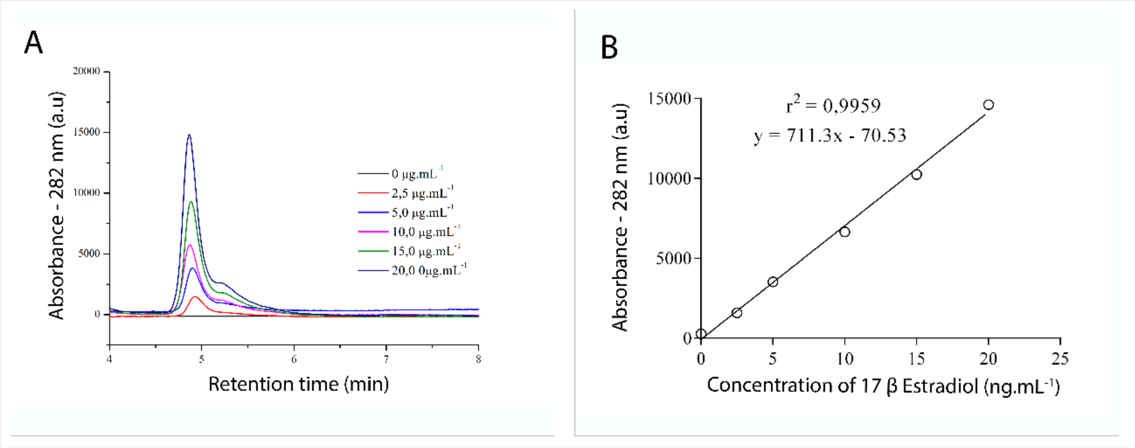

Figure S5. Chromatograms obtained for the detection of 17β-estradiol by High Performance Liquid Chromatography (A) and standard curve (B). Operating conditions: PDA detector 282 nm, Flow 0.5 mL / min, isocratic elution H_2O: ACN (60: 40%).
